# Development and Physicochemical Characterization of Edible Chitosan–Casein Hydrogel Membranes for Potential Use in Food Packaging

**DOI:** 10.3390/gels10040254

**Published:** 2024-04-09

**Authors:** Andreas Karydis-Messinis, Christina Kyriakaki, Eleni Triantafyllou, Kyriaki Tsirka, Christina Gioti, Dimitris Gkikas, Konstantinos Nesseris, Dimitrios A. Exarchos, Spyridoula Farmaki, Aris E. Giannakas, Constantinos E. Salmas, Theodore E. Matikas, Dimitrios Moschovas, Apostolos Avgeropoulos

**Affiliations:** 1 Department of Material Science and Engineering, University of Ioannina, 45110 Ioannina, Greece; pstm100417@uoi.gr (C.K.); triantafyllou.eleni@uoi.gr (E.T.); ktsirka@uoi.gr (K.T.); christina.gioti@uoi.gr (C.G.); d.exarchos@uoi.gr (D.A.E.); s.farmaki@uoi.gr (S.F.); ksalmas@uoi.gr (C.E.S.); matikas@uoi.gr (T.E.M.); 2 DODONI SA, 1 Tagmatarchi Kostaki, Eleousa, 45500 Ioannina, Greece; dgkikas@dodoni.eu (D.G.); knesseris@dodoni.eu (K.N.); 3 Hellenic Institute for Packaging and Agrifood Safety, 45445 Ioannina, Greece; 4 Department of Food Science and Technology, University of Patras, 30100 Agrinio, Greece; agiannakas@upatras.gr

**Keywords:** chitosan, casein, glycerol, dairy wastes, edible membranes, food packaging, sustainability

## Abstract

The increasing global concern over plastic waste and its environmental impact has led to a growing interest in the development of sustainable packaging alternatives. This study focuses on the innovative use of expired dairy products as a potential resource for producing edible packaging materials. Expired milk and yogurt were selected as the primary raw materials due to their protein and carbohydrate content. The extracted casein was combined with various concentrations of chitosan, glycerol, and squid ink, leading to the studied samples. Chitosan was chosen due to its appealing characteristics, including biodegradability, and film-forming properties, and casein was utilized for its superior barrier and film-forming properties, as well as its biodegradability and non-toxic nature. Glycerol was used to further improve the flexibility of the materials. The prepared hydrogels were characterized using various instrumental methods, and the findings reveal that the expired dairy-based edible packaging materials exhibited promising mechanical properties comparable to conventional plastic packaging and improved barrier properties with zero-oxygen permeability of the hydrogel membranes, indicating that these materials have the potential to effectively protect food products from external factors that could compromise quality and shelf life.

## 1. Introduction

An up-to-date food cycle, not of the food itself, consists of various important tasks that are necessary such as processing, post-treatment storage, packaging, distribution, retail, and consumption [1]. The transition from a linear to circular bio-based economy is of utmost importance, and new technologies that transform biological feedstock and/or resources into valuable products are required. The newly designed products should be renewable and cost effective to address the depletion of natural resources, the escalating global food demand and consumption, and the severe climate change [2].

In recent scientific studies, the development of bio-based materials, showcasing desired properties such as sustainability, resource efficiency and low carbon dioxide emissions have been reported [3]. The global output of plastics has reached an unprecedented value of 407 MMT (million metric tons), as already reported in the literature [4], and the COVID-19 pandemic contributed significantly to an increase in the aforementioned value. Petroleum-based plastics utilized in the packaging sector alone account for approximately 44% of the overall value, indicating their major contribution to the environmental pollution [4]. It should be mentioned though that various inherent properties, including low cost, permeability, transparency, enhanced tensile and thermal performances, and the ability to be easily sterilized, have enabled the wide use of plastic materials in packaging applications [5] so far, despite the great environmental concerns in the last couple of decades. The combination of carbon–carbon bonds in the polymeric materials, which does not allow for easy treatment and disintegration after the end-life cycle, and their excessive use have led to devastating effects on the environment, causing contamination and putting all living organisms in danger. The inability to be disintegrated in a reasonable amount of time in nature from factors such as UV irradiation leads to an accumulation of plastics in the environment as well as oceans. Approximately 8 MMT of plastics end up in the oceans each year [6]. Currently, polymeric materials comprised of polyethylene, polypropylene, and poly(ethylene terephthalate) are extensively used in food packaging. These petroleum polymers, despite their low weight and ability to be easily transformed through extrusion into variable shapes, do not showcase any sustainable characteristics. Their non-biodegradable nature contributes to the environmental pollution, as has already been mentioned. Furthermore, the utilization of synthetic polymeric materials in food packaging has detrimental effects due to the release of carbon dioxide and other toxicants during the incineration process. Hazardous interactions between food and recycled or reused plastics have also been reported in the literature [7].

The development of new packaging materials targeting food packaging applications requires in-depth analysis in terms of biodegradability. Plastics can be classified into two distinct categories, strongly dependent on their components being bio- or fossil-based. In both types, biodegradable and non-biodegradable components and their combination can be found. It should be mentioned that the final chemical structure of the materials determines their biodegradability and not the initial resource used [8]. Bioplastics are therefore categorized as bio-based and nonbiodegradable, bio-based and biodegradable, and fossil-based and biodegradable, respectively. Through this classification, it is easily understood that not all bioplastics can be completely biodegradable, and misinterpretations due to commercial purposes often occur.

The design and development of sustainable materials, which are harmonized with the current environmental concerns and exhibit high quality, are vital for both industry and consumers. The scientific community has shifted its interest toward the production of edible and biodegradable materials that adhere to food quality and safety standards [9]. Environmental contamination related to expired dairy products including milk, cheese whey, colostrum, and additional dairy industry by-products derived from the processing procedures has not gained tremendous attention yet, and only a limited number of studies have made use of these by-products to form sustainable materials [10]. The dairy by-products may possibly lead to high-quality products, further contributing to the circular economy.

In the case of food-grade components [11], bio-based, biodegradable, and edible products can be made. Edible packaging is rapidly evolving as a sustainable/biodegradable substitute of conventional packaging, demonstrating advantageous characteristics. The shelf life of edible membranes can be further extended using various additives such as lipids, chitosan/chitin, gums, cellulose derivatives, animal or plant-based proteins, and starches. Valuable characteristics involving bio-compatibility, non-toxicity, non-polluting, gas, and moisture barrier properties render the specific materials quite important for packaging-based applications [12]. Furthermore, edible materials can be designed using proteins, polysaccharides, and oils, which are derived from feedstock and active chemicals such as antioxidants and/or antimicrobial agents. These reagents are used to further enhance their final properties, making them ideal candidates in food science. The dual application of the above-mentioned materials, meaning packaging and consumption, without posing any threat to human health is important, and by tuning the thickness of the films, different characteristics for different applications can be induced. The appropriate choice of edible packaging materials is related to the potential content/food to be packed and the processing technique [13].

Substances isolated from blood and glandular fluid, namely proteins, that vary in terms of molecular weight, concentration, and function are used in such applications. The use of dairy products has been extended beyond consumption, and dairy products can be utilized in different fields as packaging [14]. Expired dairy products contribute significantly to the environmental pollution but still contain a variety of proteins that either offer defense against enteropathogens or are necessary to produce new dairy products [15]. Large amounts of proteinaceous waste, particularly whey and caseins, are produced from dairy wastes. In bovine milk, caseins constitute approximately 80% of the total protein, which makes it the most abundant type of protein [16]. Even though almost half of the whey generated globally is recovered and used in various products, including meals, supplements, and medications, huge quantities are discarded without any prior processing [17]. The inherent characteristics of milk proteins, such as high barrier and film properties, make them ideal for biomaterial synthesis [18]. In recent years, the preparation of protein hydrogel membranes from dairy waste has attracted the interest of several scientific groups. Laetitia M. Bonnaillie et al. [19] synthesized casein/glycerol/citric pectin membranes to study the structure and mechanical properties by adding a polysaccharide and a plasticizer. Muhammad Rehan Khan et al. [20] highlighted the impact of active ingredients on the composition of materials and lifespan of products, considering the presence of active ingredients in the casein matrix.

In the present study, chitosan, casein, glycerol, and squid ink were utilized for the development of films. The tested concentrations of the mixed substances and the code names were as follows: Chi_50_Cas_50_ (%wt: 50/50), Chi_33_Cas_67_ (%wt: 33/67), Chi_38_Cas_38_Gly_24_ (%wt: 38/38/24), and Chi_32_Cas_32_Gly_20_SqInk_16_ (%wt: 32/32/20/16). Chitosan was selected because of its attractive properties, such as antimicrobial properties, biodegradability, film-forming properties [21]. Casein was used because of its excellent barrier and film-forming properties, while it is also biodegradable and non-toxic [22]. Τhe combination of the two biopolymers was carried out both to combine the excellent properties of the materials and to overcome the brittle nature of the casein membrane. The squid ink was used to impart antimicrobial and antioxidant properties to the prepared films [23]. The main objective of this study was to examine the effect of casein incorporation in the materials’ mechanical, morphological, barrier properties, etc. Casein protein was isolated from expired cow milk, using the precipitation method followed by the preparation of relative hydrogel membranes under different ratios of casein. For comparison reasons, membranes of pure chitosan, chitosan, and casein as well as of chitosan, casein, and glycerol were prepared. For the final hydrogel membranes, according to the reagents used, abbreviations of the type A_x_B_y_C_z_D_w_ are used, where A, B, C, and D are the compounds (chitosan, casein, glycerol, and squid ink, respectively) and x, y, z, and w, the relative %wt ratios.

## 2. Results and Discussion

### 2.1. ATR-FTIR

**Figure 1:**
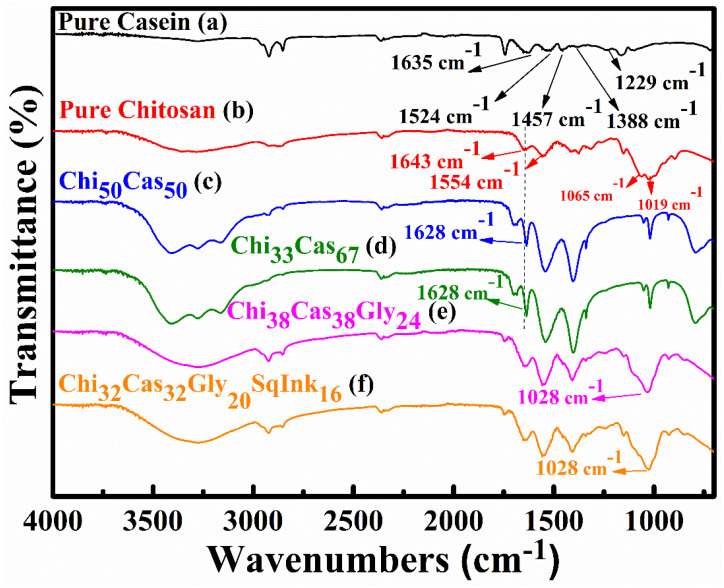
FTIR spectra of (a) pure casein, (b) pure chitosan, (c) Chi_50_Cas_50_, (d) Chi_33_Cas_67_, (e) Chi_38_Cas_38_Gly_24_, and (f) Chi_32_Cas_32_Gly_20_SqInk_16_.

ATR-FTIR spectroscopy was employed to evaluate the presence of electrostatic interactions between casein and chitosan. In Figure 1, the ATR-FTIR spectra of pure casein, Chi_50_Cas_50_ and Chi_33_Cas_67_ (chitosan/casein blend), Chi_38_Cas_38_Gly_24_ (chitosan/casein/glycerol blend), and Chi_32_Cas_32_Gly_20_SqInk_16_ (chitosan/casein/glycerol/squid ink blend) are presented. For pure casein, the FTIR spectra are shown in Figure 1a, where the broad peak observed at ~3280 cm^−1^ is attributed to -OH and -NH- stretching (the peaks are overlapping). The peaks at 2923 cm^−1^ and 2856 cm^−1^ are attributed to -CH- stretching vibrations, while the peaks observed at 1635 cm^−1^ (amide I), 1524 cm^−1^ (amide II), 1457 cm^−1^, 1388 cm^−1^, and 1236 cm^−1^ (amide III) are attributed to -C=O stretching vibrations, -NH- bending vibrations, -CH- bending vibrations (both 1457 and 1388 cm^−1^), and -NH- bending vibrations, respectively [24,25,26]. Figure 1b concerns the pure chitosan membrane, where the broad peak between 3500–3000 cm^−1^ is attributed to stretching vibrations of the -OH groups and -NH- groups (overlapping, as mentioned above). Peaks observed at 2923 and 2856 cm^−1^ are representative of -CH- stretching vibrations. The characteristic peaks at 1643 cm^−1^, 1554 cm^−1^, 1065 cm^−1^, and 1019 cm^−1^ are ascribed to -C=O stretching vibrations, -NH- bending vibrations, and -C=O stretching vibrations, respectively [27,28]. Figure 1c,d show the spectra of the Chi_50_Cas_50_ and Chi_33_Cas_67_ membranes, respectively, which are different in composition and are compared to the pure chitosan and pure casein membranes. The amide I peak observed at 1643 cm^−1^ in pure chitosan moved to 1628 cm^−1^, indicating secondary interactions between the components of the blend [29]. Figure 1e shows the spectra of the glycerol containing material, Chi_38_Cas_38_Gly_24_. The characteristic peak at 1028 cm^−1^ can be attributed to the addition of glycerol and indicates its successful integration in the blend [27]. The addition of squid ink in the blend was concluded to have no relative or drastic changes in the final FTIR spectra (Figure 1f).

### 2.2. XRD Analysis

**Figure 2:**
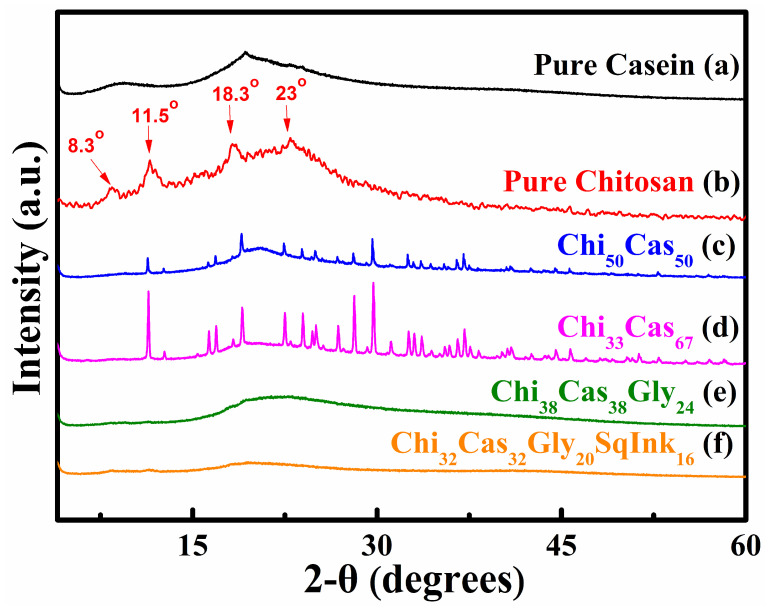
XRD diffractograms of (a) pure casein, (b) pure chitosan, (c) Chi_50_Cas_50_, (d) Chi_33_Cas_67_, (e) Chi_38_Cas_38_Gly_24_, and (f) Chi_32_Cas_32_Gly_20_SqInk_16_.

XRD analysis was utilized to examine the crystallinity of pure casein (Figure 2a), pure chitosan (Figure 2b), Chi_50_Cas_50_ (Figure 2c), Chi_33_Cas_67_ (Figure 2d), Chi_38_Cas_38_Gly_24_ (Figure 2e), and Chi_32_Cas_32_Gly_20_SqInk_16_ (Figure 2f). Proteins do not exhibit a crystalline structure in general, which means that when they are subjected to XRD analysis, normal crystalline effects do not appear [30]. The pure casein diffraction pattern is typical of nonfibrous proteins (Figure 2a). The attraction between the polar groups that most likely occur in a complex protein structure indicates the absence of structural order in casein and other similar proteins [31]. At diffraction angles 2θ of ~9.5° and 19.3°, casein displayed two flat peaks, indicating the amorphous nature of the protein. In Figure 2b, the pure chitosan diffractogram is shown and provides information of the semicrystalline nature of chitosan, and the data were already analyzed in our previous work [27]. In the diffractograms of Chi_50_Cas_50_ (Figure 2c) and Chi_33_Cas_67_ (Figure 2d), new peaks appeared, providing more evidence that intermolecular interactions between chitosan and casein occurred. The glycerol-containing materials (Figure 2e,f) were amorphous due to the high plasticizer (glycerol) concentration used [27,32].

### 2.3. TGA

**Figure 3:**
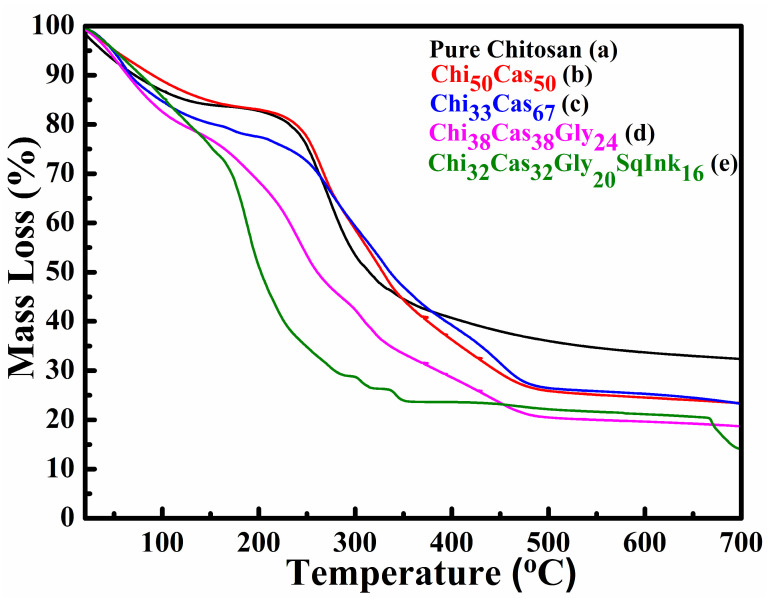
TGA thermograms of (a) pure chitosan, (b) Chi_50_Cas_50_, (c) Chi_33_Cas_67_, (d) Chi_38_Cas_38_Gly_24_, and (e) Chi_32_Cas_32_Gly_20_SqInk_16_.

In Figure 3, TGA thermograms of the membranes of pure chitosan (Figure 3a), Chi_50_Cas_50_ (Figure 3b), Chi_33_Cas_67_ (Figure 3c), Chi_38_Cas_38_Gly_24_ (Figure 3d), and Chi_32_Cas_32_Gly_20_SI_16_ (Figure 3e) are presented in a temperature range between 20 and 700 °C. The initial mass loss observed from ~80 to 120 °C is attributed to the water loss from the membranes. The incorporation of casein in the blend Chi_50_Cas_50_ did not affect the thermal stability of the membrane, while the increase in its concentration (Chi_33_Cas_67_) seems to have destabilized the material at ~180 °C. The major mass losses in the samples of pure chitosan, Chi_50_Cas_50_, and Chi_33_Cas_67_ occur from 220 to 480 °C and are ascribed to the materials’ functional group decompositions. The major losses observed in the glycerol-containing materials start at lower temperatures, which is a phenomenon that was already observed and thoroughly analyzed in our previous work [27,32]. The remaining ~20–30% of the mass is attributed to remaining ash that has been carbonized and cannot decompose any further [27,33].

### 2.4. DMA

**Figure 4:**
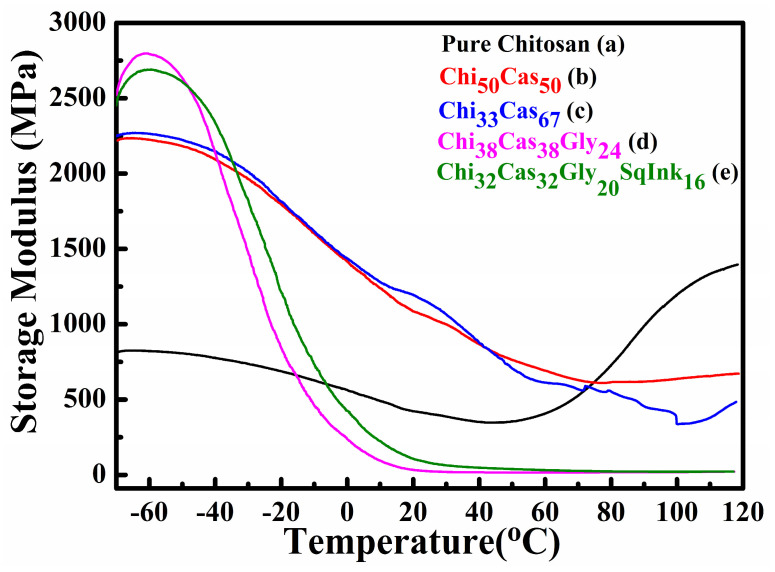
Storage modulus plots of (a) pure chitosan, (b) Chi_50_Cas_50_, (c) Chi_33_Cas_67_, (d) Chi_38_Cas_38_Gly_24_, and (e) Chi_32_Cas_32_Gly_20_SqInk_16_.

Dynamic mechanical analysis (DMA) was utilized to study the thermomechanical properties of the final membranes. In Figure 4, the results of the storage modulus as a function of temperature are illustrated for pure chitosan (Figure 4a), Chi_50_Cas_50_ (Figure 4b), Ch_33_Cas_67_ (Figure 4c), Chi_38_Cas_38_Gly_24_ (Figure 4d), and Chi_32_Cas_32_Gly_20_SqInk_16_ (Figure 4e). The thermomechanical properties of the materials were examined in the range between −70 and 120 °C. The addition of casein in the membranes Chi_50_Cas_50_ and Chi_33_Cas_67_ significantly increased the storage modulus of the materials in comparison to the pure chitosan membrane. On the other hand, the membranes Chi_38_Cas_38_Gly_24_ and Chi_32_Cas_32_Gly_20_SqInk_16_ showed decreased storage moduli due to the plasticizing effect of glycerol, while squid ink did not affect the mechanical properties.

**Figure 5:**
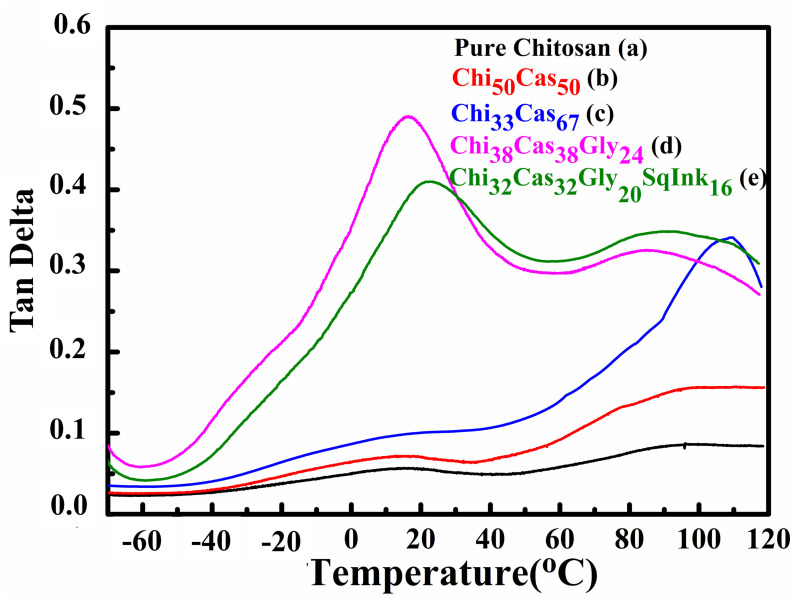
Tan delta plots of (a) pure chitosan, (b) Chi_50_Cas_50_, (c) Chi_33_Cas_67_, (d) Chi_38_Cas_38_Gly_24_, and (e) Chi_32_Cas_32_Gly_20_SqInk_16_.

In Figure 5, tan delta graphs as functions of temperature are shown. The glass transition temperature (T_g_) of pure chitosan membrane (Figure 5a) was found to be approximately 90 °C. The addition of casein in the blends led to increased T_g_s (98 °C and 108 °C, respectively) of the membranes Chi_50_Cas_50_ (Figure 5b) and Chi_33_Cas_67_ (Figure 5c). The increased T_g_ in the blends may be attributed to the T_g_ value of casein, which is higher than that of chitosan according to the literature [34], and from a 1/1 ratio of chitosan/casein in the first blend, this increased to 1/2 in the second case, explaining the 10 °C increase. The glycerol addition in the membranes Chi_38_Cas_38_Gly_24_ (Figure 5d) and Chi_32_Cas_32_Gly_20_SqInk_16_ (Figure 5e) led to a significant overall decrease (~80 °C) in the T_g_ values of the blends due to the functioning role of glycerol as a plasticizer [27,32]. The T_g_ values of the glycerol-containing materials were found to be 16 °C and 22 °C for Chi_38_Cas_38_Gly_24_ and Chi_32_Cas_32_Gly_20_SqInk_16_, respectively. It is evident that the addition of the squid ink led to a decreased ratio of glycerol in the final membrane, and therefore, the increase in 6° C in the observed T_g_ can be explained as a decrease of the plasticizer content.

### 2.5. Tensile Properties

**Figure 6:**
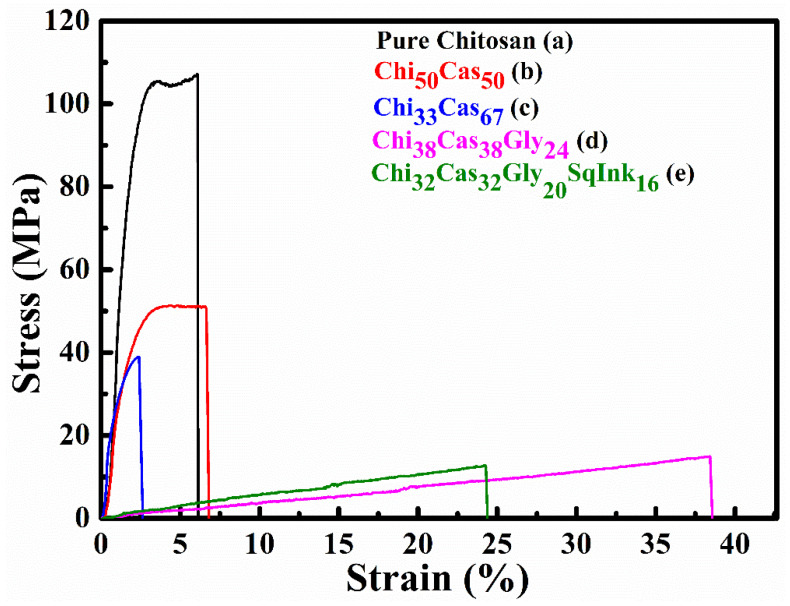
Indicative stress vs. strain plots of (a) pure chitosan, (b) Chi_50_Cas_50_, (c) Chi_33_Cas_67_, (d) Chi_38_Cas_38_Gly_24_, and (e) Chi_32_Cas_32_Gly_20_SqInk_16_.

The tensile properties of the studied membranes are depicted in the stress vs. strain plots of Figure 6, and the average stress and strain values for all samples are summarized in Table 1. The pure chitosan reference samples exhibited the highest strength of 102.82 MPa with an average strain of 6.8%. The addition of casein in the membrane at a ratio of 50% caused a reduction in the strength by 49.8% and a small increase in the strain by 13.2%, while the addition of a higher amount of 67% casein led to a further embrittlement of the specimens with reductions in both the strength and the strain values by 64.2% and 61.5%, respectively, in comparison to the reference sample (pure chitosan). The combined addition of casein and glycerol caused a significant deterioration in the strength by 85.1% with a parallel significant enhancement of the strain by 467.5% in comparison to the pure chitosan. Finally, when chitosan was mixed with casein, glycerol, and squid ink, the resulting strength was substantially reduced by 88.2%, but at the same time, the strain was increased by 250.7% compared to the pure chitosan samples. The reduced strength observed after the integration of casein in the hydrogel membrane may be attributed to poor miscibility between the blend components. Εspecially in the case of the material Ch_33_Cas_67_, in Figure 5c, a phase separation phenomenon is observed (two different Tg_s_). The integration of glycerol further reduced the strength due to the plasticizing effect that causes significantly increased strain. The binary system containing chitosan and casein showed increased values compared to the protein-based materials that are available in the scientific literature [35,36,37]. The ternary and quaternary systems Chi_38_Cas_38_Gly_24_ and Chi_32_Cas_32_Gly_20_SqInk_16_, respectively, are novel, and thus, there are no data available in the literature for comparison.

**Figure 7:**
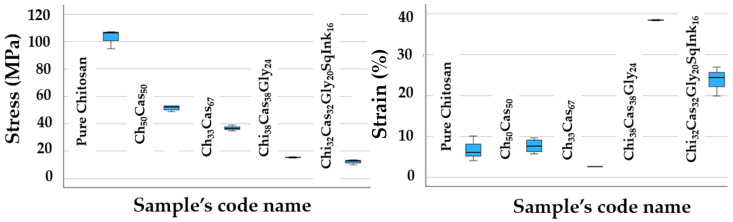
Graphical presentation of Table 1’s statistical values.

A statistical hypothesis test conducted according to the Kruskal–Wallis non-parametric method indicated as an overall conclusion that there is a significant difference between the stress and strain% mean values (Figure 7). Nevertheless, a more detail pairwise comparison provided further results, which are presented in Table 1 using superscripts.

### 2.6. SEM Measurements

**Figure 8:**
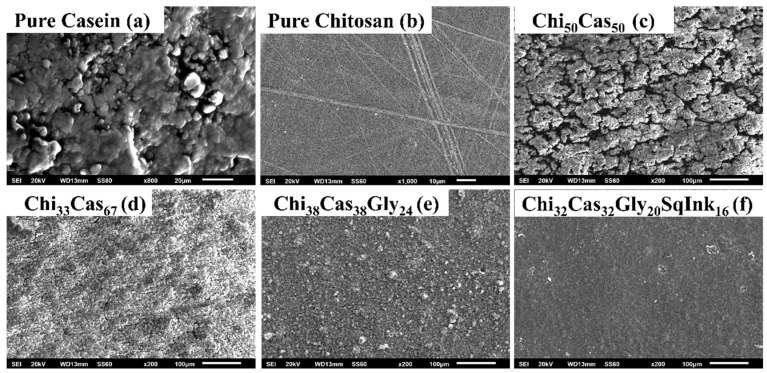
SEM images of (**a**) pure casein, (**b**) pure chitosan, (**c**) Chi_50_Cas_50_, (**d**) Chi_33_Cas_67_, (**e**) Chi_38_Cas_38_Gly_24_, and (**f**) Chi_32_Cas_32_Gly_20_SqInk_16_.

SEM was used to examine the surface morphology of the prepared membranes. Indicative SEM images of pure casein and the membranes of pure chitosan, Chi_50_Cas_50_, Chi_33_Cas_67_, Chi_38_Cas_38_Gly_24_, and Chi_38_Cas_38_Gly_24_SqInk_16_ are shown in Figure 8. In Figure 8a, the formation of a thick network is evident, composed of casein aggregates, while in Figure 8b, the dense morphology of the pure chitosan membrane is shown. In the case of the Chi_50_Cas_50_ membrane (Figure 8c), a rough surface with voids is observed, while in the case of the Chi_33_Cas_67_ membrane (Figure 8d), the surface remained rough, but the voids significantly decreased. This type of morphology could be attributed to the poor miscibility of the components. The integration of glycerol in the Chi_38_Cas_38_Gly_24_ membrane (Figure 8e), further decreased the voids on the membrane surface, and the introduction of the squid ink in the Chi_32_Cas_32_Gly_20_SqInk_16_ membrane (Figure 8f) led to a smoother and continuous dense surface morphology. The decreased presence of voids in the case of the materials Chi_38_Cas_38_Gly_24_ and Chi_32_Cas_32_Gly_20_SqInk_16_ indicates that plasticizer incorporation enhanced the miscibility between the blend’s components.

### 2.7. WVTR—Water/Vapor Diffusion Coefficient Calculation

**Figure 9:**
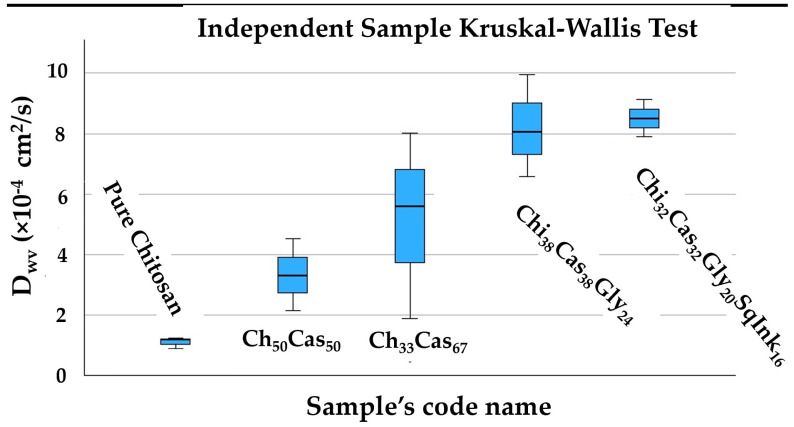
Graphical presentation of Table 2’s statistical values.

The obtained water vapor transmission rate (WVTR) values for the pure chitosan, Chi_50_Cas_50_, Chi_33_Cas_67_, Chi_38_Cas_38_Gly_24_, and Chi_32_Cas_32_Gly_20_SqInk_16_ membranes are summarized in Table 2. From these values, water/vapor diffusivity (D_w_) values were calculated and are listed in Table 2 for comparison. As seen in Table 2, the incorporation of casein in the membranes led to an increase in the D_wv_ (water/vapor diffusion coefficient) values of the obtained Chi_50_Cas_50_ film. An increase in casein content in the Chi_33_Cas_67_ film led to a higher D_wv_ value. The results could be attributed to the water-binding properties of casein [38]. The casein-containing hydrogel membranes showed higher WVPs than the pure chitosan hydrogel. The results are in agreement with the scientific literature [37], while the further increase observed in the WVP value after the addition of glycerol is normal due to the hydrophilic nature of glycerol. Squid ink further increased the D_wv_ of the Chi_32_Cas_32_Gly_20_SqInk_16_ hydrogel membrane. As an overall conclusion, we could say that the addition of casein or glycerol into the chitosan polymeric matrix led to lower water barrier. Nevertheless, according to the statistical analysis (Figure 9), pure chitosan’s mean water barrier is statistically equal to that of Chi_50_Cas_50_ or Chi_33_Cas_67_. The difference of the barrier mean value starts to be statistically significant with the addition of glycerol.

### 2.8. OTR—Oxygen Permeability Calculation

The obtained OTR values for the pure chitosan, Chi_50_Cas_50_, Chi_33_Cas_67_, Chi_38_Cas_38_Gly_24_, and Chi_32_Cas_32_Gly_20_SqInk_16_ membranes are summarized in Table 3.

The oxygen permeability studies revealed that all the prepared membranes showed zero oxygen permeability, which is one of the most significant factors about packaging materials. This result significantly strengthens the initial assumption that these materials can be used as food packaging materials. Murrieta-Martínez et al. reported that casein films showed two-times lower oxygen permeability values than whey protein concentrate films [35]. The distribution of polar amino acids throughout the protein chain endows casein hydrogel membranes with unique barrier characteristics that prevent oxygen and other non-polar molecules from penetrating the film [39]. To the best of our knowledge, the production of impermeable oxygen chitosan/casein hydrogel membranes has never been reported in the scientific literature, rendering the proposed materials with possible oxidation-prevention properties.

## 3. Conclusions

The physicochemical characterizations revealed that chitosan and casein interact through secondary interactions, while glycerol successfully integrates in the blend. Casein addition in the case of Chi_50_Cas_50_ led to a strain enhancement of 13.2%, while glycerol’s incorporation further improved the strain property of the films by 467.5%. This significant improvement in strain value makes the material easily applicable and easy to use in contrast to glycerol-free materials, which are brittle. The low glass transition temperature allows flexibility both at room temperature and at lower temperatures. The zero-oxygen permeability of the prepared films is a strong indication that they may provide effective food protection, quality assurance, and shelf-life extension. Thus, the results strongly support that the prepared novel materials can be used as sustainable packaging materials.

## 4. Materials and Methods

### 4.1. Materials

Sigma–Aldrich (St. Louis, MO, USA) was the supplier of low-molecular-weight chitosan (75–85% deacetylated), glycerol, acetic acid (99.8%), methanol (99.8%), ethanol (99.8%), sodium hydroxide (98%), and diethyl ether. The expired milk was supplied from the national dairy industry DODONI S.A., and the squid ink was purchased from a local market.

### 4.2. Extraction of Casein

For the casein extraction from expired dairy product, a beaker containing 500 mL of expired milk was heated until it reached 55 °C. In another beaker, 500 mL of acetic acid solution (approximately 10% *v*/*v*) was heated under the same conditions (temperature and time). The foam that formed on the surface of the milk was carefully removed, and the acetic acid solution was added into the beaker, dropwise, to adjust the pH at approximately 4.6, which is the isoelectric point of casein. To obtain a solid, the mixture was filtrated with filter paper. The solid sample was washed several times with distilled water and was placed in another beaker that contained enough ethanol to cover the solid. Filtration took place again with filter paper to collect the solid sample. The protein sample was washed with a 250 mL solution of ethanol/diethyl ether in a ratio of 1:1 and once more with 100 mL of diethyl ether. The extracted sample was left to dry at room temperature.

### 4.3. Membrane Synthesis

**Figure 10:**
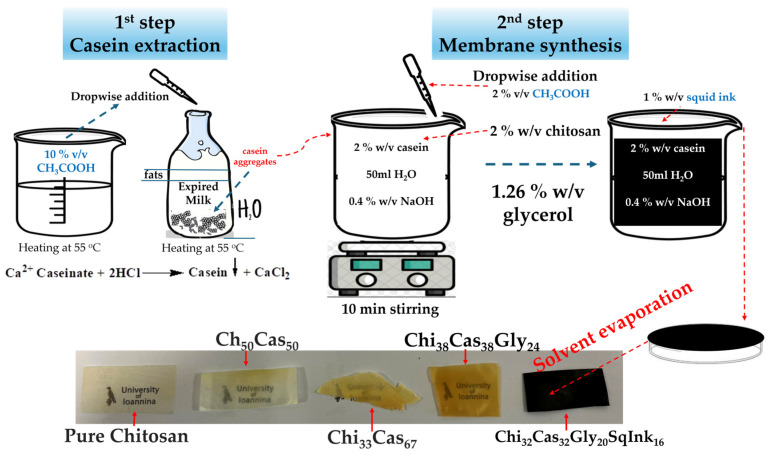
Concise schematic of membrane synthesis.

For the Chi_32_Cas_32_Gly_20_SqInk_16_ membrane synthesis, in a beaker containing 50 mL of distilled water and 2% (*w*/*v*) of extracted casein, 0.4% (*w*/*v*) of sodium hydroxide was added. The mixture was transferred to an ice bath, and it was sonicated using an ultrasonicator (UP100H, 100W, 30kHz, Hielscher Ultrasonics, Oderstraße 53, Teltow, Germany) for 5 min and then stirred until completely dissolved. While it was stirred, 2% (*w*/*v*) of chitosan was added into the solution, followed by an addition of 2% (*v*/*v*) acetic acid. The new solution was stirred for approximately 10 min. In the same beaker, 0.5 mL of glycerol was added, followed by an addition of 1% (*w*/*v*) squid ink. The final solution contained 1 g (2% *w*/*v*) of extracted casein, 1 g (2% *w*/*v*) of chitosan, 0.63 g (1.26% *w*/*v*) of glycerol, and 0.5 g (1% *w*/*v*) of squid ink. The solution was then transferred to polystyrene dishes to evaporate the solvent and form the membrane. The Petri dishes were left in room temperature overnight (around 18 h). For the preparation of all other materials, pure chitosan, Chi_50_Cas_50_, Chi_33_Cas_67_, and Chi_38_Cas_38_Gly_24_, a similar procedure was followed but without the additional step of the aforementioned method. All the samples were produced in triplicate. The abbreviations and quantities used in all the experiments are listed in Table 4. The synthesis is shown in Figure 10.

### 4.4. Attenuated Total Reflectance-Fourier Transform Infrared Spectroscopy *(ATR-FTIR)*

ATR-FTIR analysis was conducted with a SHIMADZU IRSpirit fourier transform infrared spectrophotometer (1, Nishinokyo Kuwabara-cho, Nakagyo-ku, Kyoto, Japan). The ATR objective featured a ZnSe prism with a 250 μm contact area on the studied samples. The prism allowed for a penetration depth of around 2.0 µm (@1000 cm^−1^) and enabled measurements starting from 650 cm^−1^.

### 4.5. X-ray Diffraction (XRD)

The samples’ crystallinity values were examined using a PANalytical X’PertPRO diffractometer (Enigma Business Park, Grovewood Rd, Malvern, UK) using Cu/Kα radiation. The diffractometer was equipped with an X’Celerator detector running at 40 kV voltage and 40 mA current. The membranes underwent scanning within the 2θ range from 2° to 60°.

### 4.6. Thermogravimetric Analysis (TGA)

TGA analysis was carried out utilizing a Setsys Evolution-Setaram (7, rue de l’Oratoire Caluire-et-Cuire, France) TGA, TG-DSC, and TG-DTA analyzer. The procedure involved placing roughly 30 mg of a sample into a platinum crucible, adjusting the heating and nitrogen (N_2_) flow rates, and then conducting the test. Throughout all experiments, the heating rate remained constant at 10 K/min, and the N_2_ flow rate, at 25 mL/min within the temperature range from room temperature to 700 °C.

### 4.7. Dynamic Mechanical Analysis (DMA)

The films’ dynamic mechanical behaviors were examined using a dynamic mechanical analyzer (DMA Q800, TA Instruments, 159 Lukens Drive New Castle, DE, USA) in film tension mode. To evaluate the storage modulus (E′) and the loss factor (tan δ), a temperature range from −70 °C to 120 °C at a rate of 3 K/min, along with a frequency of 1 Hz, was applied.

### 4.8. Mechanical Properties

The membranes’ tensile characteristics were assessed following ASTM D638 standards, employing a custom horizontal tensile testing stage manufactured by ADMET (51 Morgan Drive | Norwood, MA, USA). Specimens in type V dumbbell shapes were prepared and subjected to testing at a strain rate of 0.1 min^−1^ until failure. Each membrane type underwent testing at least three times by making the required type V dumbbell-shaped specimens. The elongation of these specimens was tracked using a linear variable differential transformer (LVDT), while the load was measured through a 44.5 N load cell (or a 445 N load cell for the pure specimens). The elongation values were transformed into engineering strain by dividing each specimen’s initial effective length, while the load values were transformed into engineering stress by dividing by the specimen’s cross-sectional area. The test was performed in triplicate (n = 3).

### 4.9. Scanning Electron Microscopy (SEM)

The surface morphologies of the samples were observed using a JEOL JSM-6510 LV SEM Microscope (Ltd., Tokyo, Japan) equipped with an X-Act EDS detector from Oxford Instruments, Abingdon, Oxford shire, UK (an acceleration voltage of 20 kV was applied) with a possibility to function under low-vacuum conditions. Before examination, all membranes were sputter-coated with gold/*palladium* for 45 s to prevent sample charging during observation with SEM.

### 4.10. Water Vapor Transmission Rate Measurements—Water Diffusion Coefficient Calculation

The water vapor transmission rate (WVTR [g/(cm^2^/s)]) for all the obtained membranes was calculated according to the ASTM E96/E 96M-05 method at 38 °C and 95% RH, using a custom-made apparatus. The calculated WVTR values were converted into water vapor diffusivity (D_wv_) values according to theory and relative equations, which are described in detail in a previous publication by our group [40]. The test was performed in triplicate (n = 3).

### 4.11. Oxygen Transmission Rate Measurements—Oxygen Permeability Calculation

The oxygen transmission rate (OTR) values (cc O_2_/m^2^/day) for each membrane were assessed following ASTM D 3985 standards (23 °C and 0% RH). An oxygen permeation analyzer (O.P.A., 8001, Systech Illinois Instruments Co., Johnsburg, IL, USA) was utilized for these measurements. Subsequently, in using the derived OTR values, the oxygen permeability coefficient values (PeO_2_) were calculated by employing the theoretical framework and equations described thoroughly in a prior publication by our group [40]. The test was performed in triplicate (n = 3).

### 4.12. Statistical Analysis

All data acquired from the water vapor/oxygen barrier and mechanical properties measurements were subjected to statistical analyses to indicate any statistical differences. Three different species of each kind of film were measured. The non-parametric statistical procedure of Kruskal–Wallis was used for these analyses. Overall runs as well as multiple comparison tests between all combinations of sample couples were carried out. For all groups of experimental measurements, a statistical analysis interpretation was carried out assuming a significance level of *p* < 0.05 and using the SPSS software (v. 29.0.1, IBM, Armonk, NY, USA).

## Figures and Tables

**Table 1:** Mechanical properties of the prepared membranes.

Specimen	Stress (MPa)	Strain (%)	Elongation at Break (mm)	% Change in Stress *	% Change in Strain *
**Pure Chitosan**	102.82 ± 6.97 ^a^	6.77 ± 3.01 ^a^	1.79 ± 0.79	Reference system	
**Chi_50_Cas_50_**	51.60 ± 2.00 ^a,b^	7.66 ± 1.72 ^a^	2.01 ± 0.40	−49.82	+13.15
**Chi_33_Cas_67_**	36.84 ± 2.02 ^a,b,c^	2.61 ± 0.07 ^a^	0.69 ± 0.02	−64.17	−61.45
**Chi_38_Cas_38_Gly_24_**	15.36 ± 0.45 ^b,c^	38.42 ± 0.16 ^b^	10.34 ± 0.22	−85.06	+467.50
**Chi_32_Cas_32_Gly_20_SqInk_16_**	12.18 ± 1.85 ^c^	23.74 ± 3.56 ^b^	6.33 ± 1.09	−88.15	+250.66

* with respect to the reference system (Pure Chitosan). ^a–c^: Different letters in each column indicate statistically significant differences at the confidence level *p* < 0.05 (number of repetitions, n = 3).

**Table 2:** Water vapor transmission rate (WVTR) and water/vapor diffusivity (D_wv_) of the membranes: pure chitosan (a), Chi_50_Cas_50_ (b), Ch_33_Cas_67_ (c), Chi_38_Cas_38_Gly_24_ (d), and Chi_32_Cas_32_Gly_20_SqInk_16_ (e).

Samples	WVTR [×10^−7^ gr/(cm^2^*s)]	D_wv_ (×10^−4^ cm^2^/s)
Pure Chitosan	7.69367 ± 0.64878	1.10 ± 0.198 ^a^
Chi_50_Cas_50_	9.55637 ± 4.27094	3.33 ± 0.790 ^a,b^
Chi_33_Cas_67_	7.79506 ± 5.03817	5.16 ± 1.040 ^a,b^
Chi_38_Cas_38_Gly_24_	19.2141 ± 3.35570	8.20 ± 1.690 ^b^
Chi_32_Cas_32_Gly_20_SqInk_16_	15.3178 ± 3.21761	8.51 ± 0.876 ^b^

^a,b^: Different letters in each column indicate statistically significant differences at the confidence level *p* < 0.05 (number of repetitions, n = 3).

**Table 3:** Oxygen permeability calculation results of the membranes of pure chitosan (a), Chi_50_Cas_50_ (b), Chi_33_Cas_67_ (c), Chi_38_Cas_38_Gly_24_ (d), and Chi_32_Cas_32_Gly_20_SqInk_16_ (e).

Samples	OTR (mL.*m^−2^*day^−1^)	Pe_O2_ (cm^2^/s)
Pure Chitosan	<0.5 *	impermeable
Chi_50_Cas_50_	<0.5 *	impermeable
Chi_33_Cas_67_	<0.5 *	impermeable
Chi_38_Cas_38_Gly_24_	<0.5 *	impermeable
Chi_32_Cas_32_Gly_20_SqInk_16_	<0.5 *	impermeable

* According to the instrumental requirements, the calibration O.T.R. value is 0.5–0.6. The recorded values lower than 0.5 mL.m^−2^.day^−1^ are considered zero (number of repetitions, n = 3).

**Table 4:** Abbreviations and compositions of the prepared membranes.

Sample Code	Chitosan (*w*/*v* %)	Casein (*w*/*v* %)	Glycerol (*w*/*v* %)	Squid Ink (*w*/*v* %)
**Chi_32_Cas_32_Gly_20_SqInk_16_** **(%wt: 32/32/20/16)**	2	2	1.26	1
**Chi_38_Cas_38_Gly_24_** **(%wt: 38/38/24)**	2	2	1.26	-
**Chi_33_Cas_67_** **(%wt: 33/67)**	2	4	-	-
**Chi_50_Cas_50_** **(%wt: 50/50)**	2	2	-	-
**Pure Chitosan** **(%wt: 100)**	2	-	-	-
**Pure Casein** **(powder)**	-	100	-	-

## Data Availability

The datasets generated for this study are available upon request to the corresponding author.
